# Serological Survey on the Occurrence of *Rickettsia* spp., *Neospora caninum*, *Bartonella henselae* and *Toxoplasma gondii* in Cats from Tuscany (Central Italy)

**DOI:** 10.3390/ani11061842

**Published:** 2021-06-21

**Authors:** Valentina Virginia Ebani, Simona Nardoni, Michela Maestrini, Stefania Perrucci, Francesca Mancianti

**Affiliations:** 1 Department of Veterinary Sciences, University of Pisa, Viale delle Piagge 2, 56124 Pisa, Italy; simona.nardoni@unipi.it (S.N.); michela.maestrini@phd.unipi.it (M.M.); stefania.perrucci@unipi.it (S.P.); francesca.mancianti@unipi.it (F.M.); 2 Centre for Climate Change Impact, University of Pisa, Via del Borghetto 80, 56124 Pisa, Italy

**Keywords:** cats, indirect immunofluorescence test, *Neospora caninum*, *Rickettsia felis*, *Rickettsia conorii*

## Abstract

**Simple Summary:**

Domestic and stray cats are frequently infected by pathogens, some of which are zoonotic. The occurrence of microorganisms such as *Bartonella henselae* and *Toxoplasma gondii*, even though they are well known, could be underestimated because they are not regularly investigated in cats. Other pathogens, such as *Neospora caninum* and *Rickettsia* spp., have not been largely studied in feline populations and data about their spreading in Italian cats are very scanty. Monitoring of domestic and stray cats for these pathogens is important to evaluate the health status of the animals, but also, regarding *B. henselae*, *Rickettsia* spp. and *T. gondii*, from a One Health perspective.

**Abstract:**

Asymptomatic cats often harbor pathogens, some of which have not been largely investigated in feline populations. The aim of this study was to evaluate the occurrence of antibodies against *Rickettsia conorii*, *Rickettsia felis*, *Rickettsia typhi*, *Neospora caninum*, *Bartonella henselae* and *Toxoplasma gondii* in cats from Tuscany. Ninety-five blood serum samples, previously collected, were analyzed by indirect immunofluorescence assay. Fifty-six (58.94%) cats had antibodies to at least one investigated pathogen: 28 (29.47%) cats were positive for *B. henselae*, 17 (17.89%) for *R. felis*, 14 (14.73%) for *R. conorii*, 14 (14.73%) for *T. gondii*, 2 (2.1%) for *N. caninum*. No cats were positive for *R. typhi*. Positive reactions to two or more pathogens were detected in 18 (18.94%) cats. The occurrence of antibodies against these microorganisms suggests that cats, even though asymptomatic, may be infected by pathogens, often zoonotic, and thus may be a source of infections for other animals and humans.

## 1. Introduction

Domestic and stray cats (*Felis catus*), even though asymptomatic, may harbor different pathogens that can represent a relevant issue for the health status of animals and humans. Cats living outdoors have more occasions to contract microorganisms excreted by infected animals, as well as increased exposure to hematophagous arthropods that frequently act as vectors of different pathogens. Some infections, such as those caused by *Bartonella henselae* and *Toxoplasma gondii*, have been largely studied and the human clinical forms well described. On the other hand, some microorganisms can be considered as emerging pathogens and data about their circulation in feline populations, as well as the zoonotic potential for some of them, are scanty.

*B. henselae* belongs to the genus *Bartonella* which includes several species able to infect animals and humans. *B. henselae* is a facultative intracellular, hemotropic Gram-negative bacteria; it is usually transmitted by *Ctenocephalides felis*, whereas cats act as reservoirs [1]. This pathogen is the causative agent of cat scratch disease (CSD), which affects human beings, mainly children and immunocompromised patients, and is linked to conditions such as lymphadenopathy, bacteremia, Parinaud’s oculoglandular syndrome, peliosis hepatis, endocarditis, neuroretinitis, meningitis, pneumonia, musculoskeletal manifestation; bacillary angiomatosis has been related to *B. henselae* infection, too [2].

*Toxoplasma gondii* is an Apicomplexan protozoon, infecting a wide range of animals, with a life cycle that involves cats as final hosts. Infected cats can excrete unsporulated *T. gondii* oocysts by feeding on infected prey, acting as definitive hosts, or may become infected by ingesting sporulated oocysts from the environment. Asexual multiplication occurs in a wide range of intermediate hosts, among which are human beings. Toxoplasmosis is a zoonotic disease, life threatening in immunodeficient human patients and in fetuses [3] and the most common causative agent of chorioretinitis [4] in immunocompetent people, too. Moreover, *T. gondii* can induce abortion and neonatal mortality in ruminants [5]. For these reasons this infection in cats remains a public health and veterinary concern [6].

Genus *Rickettsia* includes several species able to cause disease in animals and humans, classified in the spotted fever group (SFG) and typhus group (TG).

Among SFG species, *R. conorii* is the most known rickettsial agent in Europe, responsible for the disease called Mediterranean spotted fever. The brown dog tick *Rhipicephalus sanguineus* is the main vector but also the main reservoir since dogs can suffer the illness [7]. Other SFG *Rickettsia* spp. have been found in animals and arthropod specimens in Europe, including Italy [7].

*R. felis* is considered as an emerging SFG species which infects cats and humans. It is usually transmitted by *C. felis* fleas [8]. *C. felis* is considered the reservoir of *R. felis*, which is vertically transmitted to successive generations of fleas [9], whereas cats are not considered as reservoirs of this agent because it seems that they usually develop a short-term bacteremia. *R. felis* causes human disease with common signs such as fever, headache, myalgia, cutaneous rash; furthermore respiratory, digestive and neurological symptoms have been reported [10].

*R. typhi* is a worldwide flea-borne TG rickettsial species. It is primarily transmitted by the rat flea *Xenopsylla cheopis* and rodents (mainly *Rattus norvegicus* and *Rattus rattus*) act as the main reservoirs. Cats and *C. felis* are involved in the epidemiology of this infection, too. *R. typhi* is the etiologic agent of the human disease murine typhus. Fever, headache, arthralgia and cutaneous rash are the common clinical signs in infected people, but severe disease with pneumonia and ophthalmic complications has been observed mainly in elderly and transplanted patients [11].

*Neospora caninum* is an Apicomplexan protozoon with a life cycle like *T. gondii*, but involving dogs as final hosts. *N. caninum* is responsible for abortion in bovine and in small ruminants and for neuromuscular disease in dogs. Cats are susceptible to experimental infection [12,13] as several domestic and wild animal species are exposed to the parasite [14].

Data about the circulation of *Rickettsia* spp. and *N. caninum* in Italian feline populations are very scanty, thus the aim of the present study was to verify the occurrence of antibodies against *R. felis*, *R. typhi*, *R. conorii* and *N. caninum* in clinically healthy cats living outdoors in Tuscany (Central Italy). Furthermore, the serum samples were also submitted to serological tests for *B. henselae* and *T. gondii*.

## 2. Material and Methods

### 2.1. Animals

Ninety-five blood serum samples previously collected from cats were selected and used in this survey. The samples were collected from January 2018 to March 2021 from cats during routine clinical visits for FIV/FeLV screening by collaborating veterinarians. Owners’ consent was obtained in all cases. Among the available serum samples, only those from cats with the following features were used in the present investigation: (1) FIV/FeLV negative, (2) clinically healthy, (3) living outdoors, (4) infested by fleas and/or ticks, (5) not under antibiotic treatment.

The 95 selected animals were 35 females and 60 males; 47 were ≤12 months old and 48 >12 months old.

### 2.2. Serological Tests

Indirect immunofluorescence assay (IFA) was carried out, following the manufacturers’ instructions, to detect antibodies against the investigated antigens. The tests were executed using different commercial IFA slides coated with the following antigens: *B. henselae*, *N. caninum*, *R. conorii*, *R. felis*, *R. typhi* (Fuller Laboratories Fullerton, Fullerton, CA, USA) and *T. gondii* (Toxospot IF^®^ Biomérieux, Lyon, France), respectively. A fluorescein isothiocyanate-conjugated sheep anti-cat IgG (Sigma-Aldrich, Saint Louis, MO, USA) diluted 1:50 in Evans Blue (Sigma-Aldrich, Saint Louis, MO, USA) solution was used.

All sera were tested at 1:32 dilution for *B. henselae* and rickettsiae and at 1:20 for *T. gondii* and *N. caninum* [15]. Positive samples were twofold serially diluted to determine the endpoint titer.

### 2.3. Statistical Analysis

Statistical evaluation was carried out by the χ^2^ test to analyze the results of serological tests in relation to gender and age. Values of *p* < 0.05 were considered significant.

## 3. Results

The 95 tested cats were infested by *C. felis* fleas. Fifty-six (58.94%) cats had antibodies to at least one investigated pathogen: 22/35 (62.85%) females and 29/60 (48.33%) males; 27/47 (57.44%) were ≤12 months old and 24/48 (50%) > 12 months old. The observed differences were not statistically significant.

Regarding the investigated pathogens, 28 (29.47%) cats were positive for *B. henselae* (*n* = 12 titer 1:64, *n* = 9 titer 1:128, *n* = 4 titer 1:256, *n* = 3 titer 1:512), 17 (17.89%) for *R. felis* (*n* = 7 titer 1:32, *n* = 7 titer 1:64, *n* = 3 titer 1:128), 14 (14.73%) for *R. conorii* (*n* = 11 titer 1:32, *n* = 3 titer 1:64), 14 (14.73%) for *T. gondii* (*n* = 1 titer 1:40, *n* = 4 titer 1:80, *n* = 6 titer 1:160, *n* = 1 titer 1:320, *n* = 2 titer 1:2560), 2 (2.1%) for *N. caninum* (titer 1:20). No cats were positive for *R. typhi*.

Positive reactions to two or more pathogens were detected in 18 (18.94%) cats (Table 1).

## 4. Discussion

The results obtained in the present study show that the selected feline population was exposed to most of the investigated pathogens. No statistically significant differences were observed in relation to age and gender.

A 29.47% prevalence of cats seropositive to *B. henselae* confirms this microorganism as one of the most widespread zoonotic agents in feline populations. Previous serological surveys found different percentages of positive cats, mainly in relation to the condition of the tested animals and the geographic areas where they lived. In Northern Italy, prevalences of 30% and 39% were detected in domestic and stray cats, respectively [16], whereas a later study found 23.1% of seropositive stray cats [17]. Recently, a 45.9% seroprevalence was detected in stray cats from Emilia-Romagna [18]. Values ranging between 16% and 34% were found in cats in Central Italy [19,20]. Finally, prevalences of 54.8%, 45.7% and 47.3% were detected in cats from Southern Italy [21,22,23]. Furthermore, Pinna Parpaglia et al. [24] found 22% of cats were positive in Sardinia, whereas Zobba et al. [25] found a 11% seroprevalence, with 6/55 cats positive, in the same region.

All cats tested in this survey were infested by *C. felis,* which is known as the main vector of *B. henselae*. Our findings confirm that cats parasitized by *C. felis* are often infected by *B. henselae*, suggesting that owners of these animals have a high possibility to contract this agent.

*C. felis* is also involved in the epidemiology of *R. felis*. This is an emerging zoonotic pathogen that has been found in Italy, as shown by a molecular study carried out in fleas collected in Sicily (Southern Italy) that found *R. felis* DNA in 25.76% of *C. felis* analyzed [26].

Serological studies about the prevalences of cats positive to *R. felis* and/or *R. conorii* in Italy are not numerous; thus it is difficult to compare our results with the Italian epidemiological status. Morganti et al. [27] found 8.04% and 6.29% of cats positive for *R. felis* and *R. conorii*, respectively, with low titers (1:64–1:128), whereas values of 2.4% for *R. felis* and 54.8% for *R. conorii* were observed in a small number of cats in Southern Italy [21]. A 10.8% seroprevalence of domestic cats positive to *R. felis* was detected in Central-Southern Italy [28].

In our investigation, a prevalence of 17.89% for *R. felis* was detected when cats with antibody titers >1:32 were considered as positive; considering the titer 1:64 as cutoff, the seroprevalence was 10.52%, which is more similar to the values found by both Morganti et al. [27] and Morelli et al. [28]. Furthermore, antibody titers not >1:128 were observed in our investigation, too.

Conversely, our results are not in agreement with the previous study in Southern Italy that found 54.8% of *R. conorii*-positive cats. In fact, the present survey detected 14.73% of cats positive to this agent with antibody titers >1:32, but only 3.15% with 1:64 titers. This low prevalence could be related to scarce occasions for the tested cats to contract *R. conorii*, which is usually transmitted by ticks, mainly *R. sanguineus*. In fact, the presence of ticks on cats is not frequent due to their grooming habits. However, *R. conorii* has been detected in *C. felis*, too [29], thus it cannot be excluded that cats infested by fleas can contract this SFG *Rickettsia*.

Four animals were positive to both *R. conorii* and *R. felis*; these results could be related to coinfections by the two agents or to cross-reactions that are possible between SFG rickettsias. In fact, IFA is usually employed in the serological diagnosis of rickettsiosis, being considered the gold standard method, but it cannot always discriminate infections caused by different *Rickettsia* spp. [30].

No *R. typhi*-positive cats were detected according to a previous investigation that did not find seropositive cats in Spain [31]. Conversely, 15.8% seroprevalence in naturally infected cats was found in a study carried out in Northeast Spain; moreover, in the same investigation, five seropositive cats and 55% of *C. felis* analyzed were PCR-positive for *R. typhi* [32].

In recent years, several cases of human *R. typhi* infection have been reported in the Mediterranean area [33]. In Italy, murine typhus was the most widespread rickettsiosis during World War II, mainly in Sicily, from where other cases were reported in the late 1980s. A case of murine typhus was observed in an older woman from Calabria (South Italy) in 2013 [34]. To the best of our knowledge, only one serological study investigated the occurrence of anti-*R. typhi* antibodies in cats in Italy: Morelli and coworkers [28] found 4.2% positivity in domestic cats living in some areas of Central and Southern Italy. However, all *R. typhi*-positive cats had antibodies to *R. felis*, too; thus the findings could be due to a multiple infection with these two pathogens but also to cross-reactions.

The cases of *R. typhi* infection in human beings and the epidemiological situation in the Mediterranean countries suggest the need to monitor feline populations and their fleas for this emerging rickettsia, as well as other arthropod-borne pathogens. Climate change in recent decades has favored increasing rates of hematophagous arthropods, including fleas, which may transmit several pathogens.

Two out of 95 examined cats (2.1%) scored positive for *N. caninum* with a low antibody titer. In the present report, IFA was employed, being considered as the gold standard for diagnostic purposes in many animal species [35]. However, most epidemiological studies have been carried out on bovine and canine sera.

Data about seroprevalence in cats are quite variable, so our data can be compared with the results obtained elsewhere. Even though clinical cases of feline neosporosis in naturally infected cats have not been reported, the health status of tested populations could affect seroprevalence rates, with a broad range of seropositivity. To the best of our knowledge, only five serologic studies have been carried out in Europe. Ferroglio et al. [36] found 24.8% of cats from Turin were positive, with very high antibody titers; otherwise, the seroprevalences reported by other authors would agree with our data. A serologic survey in Hungary reported 0.6% of cats positive [15]; seroprevalence of 3.86% in the Czech Republic has been thereafter found [37], as well as 0.7% in Albania [14] and 6.8% in Majorca [38]. The first report of occurrence of *N. caninum* antibodies in cats (11.9%) was from Brazil [39], then other studies have followed, with seroprevalences of 0% [40], 2.9% [41], 24.5% [42] and 37% [43]. Other reports from Thailand and Iran refer to 0% [44] and 14–19% [45], respectively. These striking differences among the studies would probably depend on the presence of final hosts in the environment, too. Dogs are responsible for the infection of rodents, which are common prey of cats living outdoors. This hypothesis is corroborated by the results of serological studies carried out in Tuscany on autochthonous wildlife mammals; they showed seroprevalences of 28% in red deer [46] and 1.3% in hares [47], from areas where several dogs were living and in protected areas not attended by them, respectively.

The rate of seroprevalence versus *T. gondii* (14.73%) appears quite low in comparison with a previous study in colony cats from Tuscany, showing 44% prevalence [48]. However, serological data of cats from Europe yielded percentages of seropositivity ranging from 10% in Persian cats from Spain [49] to 82% in domestic cats from Poland [50]. Furthermore, the data from the present study agree with the seroprevalence values of toxoplasma infection of cats from Italy, ranging from 0.4% [51] to 50.4% [52]. It is well known that the prevalence of toxoplasma infection appears to widely vary depending on the age of host, the lifestyle and the availability of food [6]. So, the present data, coupled with the low occurrence of antibodies against *N. caninum*, would suggest that the selected cats (mostly young) although living outdoors, were not used to feeding on their prey.

## 5. Conclusions

Cats are frequently infected by pathogens, most of which are zoonotic. Emerging microorganisms, such as *Rickettsia* spp., seem to circulate among feline populations through fleas and/or ticks acting as vectors. For these reasons, cats, mainly pets, should be periodically monitored to verify possible infections due not only to well-known pathogens such as *B. henselae* and *T. gondii*, but also to emerging microorganisms. Moreover, careful controls against hematophagous arthropods on cats and in domestic environments are necessary to reduce the chance of infection for cats’ owners.

## Figures and Tables

**Table 1:** Details of the cats testing positive to two or more pathogens.

Cat ID	*Bartonella henselae*	*Neospora caninum*	*Rickettsia conorii*	*Rickettsia felis*	*Toxoplasma gondii*
M11				1:32	1:2560
M14	1:64				1:2560
M26			1:64	1:64	
M23			1:32	1:64	
M27	1:128				1:160
M28	1:64	1:20		1:128	
M40	1:256			1:64	
M41			1:32	1:128	
M46			1:32		1:160
M48	1:64	1:20		1:128	
M54	1:128				1:320
PI16	1:64			1:32	
PI24				1:32	1:80
PI31	1:64		1:32		
PI45	1:128				1:40
N50	1:512		1:32	1:32	
N60	1:512		1:64		
N64	1:128		1:32		

Legend. ID: identification number.

## Data Availability

Data are contained within the article.
